# Depletion of transit amplifying cells in the adult brain does not affect quiescent neural stem cell pool size

**DOI:** 10.1186/s12576-023-00876-2

**Published:** 2023-09-13

**Authors:** Zakiyyah Munirah Mohd Zaki, Anri Kuroda, Naoko Morimura, Yoshitaka Hayashi, Seiji Hitoshi

**Affiliations:** 1 Department of Integrative Physiology, Shiga University of Medical Science, Seta Tsukinowa-Cho, Otsu, Shiga 520-2192 Japan; 2 Department of Ophthalmology, Shiga University of Medical Science, Otsu, Japan

**Keywords:** Lentiviral barcoding, Clonal analysis, Subependymal zone, Neurospheres, Transit amplifying cell, Ara-C

## Abstract

Neural stem cells (NSCs) are maintained in the adult mammalian brain throughout the animal’s lifespan. NSCs in the subependymal zone infrequently divide and generate transit amplifying cells, which are destined to become olfactory bulb neurons. When transit amplifying cells are depleted, they are replenished by the quiescent NSC pool. However, the cellular basis for this recovery process remains largely unknown. In this study, we traced NSCs and their progeny after transit amplifying cells were eliminated by intraventricular infusion of cytosine β-D-arabinofuranoside. We found that although the number of neurosphere-forming NSCs decreased shortly after the treatment, they were restored to normal levels 3 weeks after the cessation of treatment. More importantly, the depletion of transit amplifying cells did not induce a significant expansion of the NSC pool by symmetric divisions. Our data suggest that the size of the NSC pool is hardly affected by brain damage due to antimitotic drug treatment.

## Background

Quiescent neural stem cells (NSCs) are present in the subependymal zone (SEZ) of the postnatal brain in rodents and humans and maintained throughout life [1, 2]. However, the contribution of NSCs to olfactory bulb neurogenesis in humans remains controversial [3, 4]. Multipotent and self-renewing capabilities of NSCs are evidenced by several immunohistochemical and cell culture methods, including monolayer culture and a colony-forming neurosphere assay [5–8]. Although adult neurogenesis is considered relatively robust, the number of new neurons in the olfactory bulb or the dentate gyrus of the hippocampus is reduced by psychosocial and physical stress, suggesting that this process plays a significant role in the regulation of mood [9, 10]. In addition, the number of NSCs in the SEZ gradually decreases with age [11–13]. Questions remain with regard to how quiescent NSCs are lost in relation to their cell division, the generation of transit amplifying cells, immediate progeny of NSCs, and subsequent neurogenesis mechanisms.

Recent progress in lineage-tracing methodologies and single cell transcriptome analysis has provided fundamental information on the clonal propagation and biological properties of tissue stem cells [14–16]. Using highly variable barcodes introduced by a retrovirus or lentivirus in combination with a neural precursor cell-specific Cre-loxP system, lineages of individual NSCs have been analyzed to reveal that NSCs are regionally specified before embryonic day (E) 15.5 to supply neurons to different regions of the mouse brain [17]. However, one caveat to be considered for the usage of exogenously transduced barcoding is the biased amplification and overrepresentation of viruses with unique tags, leading to “lumping” errors in which cells with the same barcode are derived from independent NSCs that happen to be infected with separate viruses carrying the same barcode. To avoid these issues, we have developed a method of GFP-expressing lentivirus-mediated labeling of quiescent NSCs in postnatal mouse brains [13]. Lentivirus-infected NSCs in the adult brain can be later isolated using the neurosphere assay with clonal culture conditions, and lentiviral integration sites can be recognized as unique barcodes without “lumping” errors. Using this technology, we investigated the effects of depletion of transit amplifying cells, representing the immediate progeny of self-renewing NSCs, on the behavior of NSCs.

## Methods

### Preparation of EGFP‐expressing lentivirus

Lentiviral plasmid vector pCL20c‐MSCV‐GFP-2LoxP was generated by inserting two LoxP sequences into 3’ to the GFP of pCL20c‐MSCV‐GFP and the detailed procedures for vesicular stomatitis virus‐G protein pseudotyped lentivirus production have been previously described [13].

### Animals and lentivirus infection

The Animal Care and Use Committee of Shiga University of Medical Science approved the use and care of animals in this study. All animals were maintained at 24 °C on a 12-h light–dark cycle with free access to water and food. *Nestin*-*CreER* transgenic mice [18] and Ai14 knockin mice (Rosa-CAG-LSL-tdTomato-WPRE, #007909, Jackson Lab) on a C57 BL/6 J genetic background were maintained by mating with C57 BL/6 J mice. (Japan SLC, Japan). DNA was obtained from the tip of the tail and transgenic mice were genotyped by PCR as described [18]. Littermate wild-type mice were used in this study except as otherwise described.

Newborn pups were anesthetized by hypothermia. One μl of lentivirus solution containing 1.0 × 10^5^ TU virus and 0.1% Fast Green was injected stereotactically into the right lateral ventricle using a Hamilton syringe with a 30G needle. The pups were gently warmed to restore normal body temperature and returned to the dam. Tamoxifen (Sigma-Aldrich) was dissolved in corn oil (Nacalai Tesque, Japan) at 50 mg/ml and two doses of 10 mg tamoxifen was administered every other day by oral gavage to 6-week-old mice. Mice were injected with 5-bromo-2’-deoxyuridine (BrdU; Sigma-Aldrich; 50 mg/kg, i.p.) every 3 h for a total of five injections and sacrificed 1 h after the last injection.

### Micro-osmotic pump insertion

Twenty-week-old adult mice were anesthetized with 60 mg/kg ketamine and 12 mg/kg xylazine in PBS and a micro-osmotic pump cannula (model 1007D, Alzet) was implanted at a site 4.2 mm anterior to lambda, 0.8 mm lateral, and 2.7 mm below the surface of the skull. The cannula was fixed to the skull by dental cement (Tokuyama Dental, Japan). PBS or 2% cytosine β-D-arabinofuranoside (Ara-C; Sigma-Aldrich) in PBS [19, 20] was infused by the micro-osmotic pump to the right lateral ventricle at a rate of 0.5 μl/hour for 7 days.

### Neurosphere assay

The detailed protocol used to generate neurospheres in vitro from adult brains has been described previously [21]. Single cells from the lateral and medial portion of the lateral ventricles were cultured in 24-well and 6-well plates at a density of 5 cells/μl in serum-free media (SFM) containing 10 ng/ml FGF-2, 2 μg/ml heparin, and 20 ng/ml EGF (all from Sigma-Aldrich). After 6 days in vitro, the number of floating spherical colonies (neurospheres) possessing a diameter greater than 0.1 mm was counted by measuring the diameter with a scale equipped in an eyepiece lens. The total number of neurosphere-forming NSCs in the adult brain was calculated from the following: volume in which SEZ cells were suspended (usually 600 μl), volume applied in the culture, and number of resultant neurospheres in the 24-well plate culture.

Primary single neurospheres expressing GFP were picked up under fluorescent microscopy and mechanically dissociated into single cells in 0.2 ml of SFM containing FGF-2, heparin, and EGF, and then subcloned in 48-well (0.3 ml/well) plates (Falcon). Resultant GFP^+^ neurospheres were serially passaged and amplified in larger dishes until a sufficient number of neurospheres were obtained (usually 3 passages). Genomic DNA was extracted by proteinase K treatment.

In this study, we define quiescent NSCs as cells in the SEZ that respond to FGF-2 and EGF and proliferate to form neurospheres in vitro. On the other hand, dormant NSCs are defined as cells that retain potential to convert to quiescent NSCs but cannot be detected by the neurosphere assay.

### Inverse PCR

The protocol of inverse PCR has been previously described [13]. Briefly, genomic DNA from clonally amplified GFP^+^ neurospheres was digested with 4-base cutter restriction enzymes. DNA fragments were self-ligated with T4 DNA ligase (Takara Bio) and the resultant DNA mixture was subjected to nested PCR. We used a pair of primers, LTR-A1 5'-TGTGTGCCCGTCTGTTGTGTGA-3' and LTR-B2 5'-AGCCAGAGAGCTCCCAGGCTCA-3', for the first amplification and another pair of primers, LTR-A2 5'-TGGTAACTAGAGATCCCTCAGA-3' and LTR-B1 5'-GATCTGGTCTAACCAGAGAGAC-3', for the second PCR. Amplified DNA fragments were sequenced to determine the lentiviral integration site. Two integration site-specific primers from the mouse genome were synthesized, and nested PCR was carried out with the appropriate primers within the lentiviral DNA to detect the presence of lentiviral DNA at the integration site.

### Histochemistry

Mice receiving lentivirus and micro-osmotic pump insertion were sacrificed 10 days or 4 weeks after the surgery by deep anesthesia and transcardial perfusion of 4% paraformaldehyde. Coronal cryosections of 20-µm thickness were immunostained for GFP, GFAP and Nestin. An antigen retrieval method by irradiating the sections in a microwave for 5 min in 10 mM citrate buffer (pH 6.0) was used to immunostain BrdU. We used rabbit anti-GFP (Invitrogen; 1:2000), rabbit anti-GFAP (Dako; 1:2000), rat anti-Nestin (Fuji Film; 1:200) and mouse anti-BrdU (Millipore; 1:1000) primary antibodies, followed by appropriate Alexa Fluor-conjugated secondary antibodies (Invitrogen; 1:4000) and Hoechst 33342 (1 µg/ml; Sigma-Aldrich). Images were obtained with an Olympus microscope (Olympus BX51; Olympus) and digital camera system (Olympus DP70; Olympus) or a confocal laser-scanning microscope (SP-8; Leica) with a × 20 objective lens. Total number of BrdU^+^ cells in the lateral and medial SEZ within 400 μm rostral to the crossing of the anterior commissure was calculated by counting positive cells in 4–6 sections within the regions of interest.

### Calculation of NSC consumption probability by age

Means of total numbers of neurosphere-forming NSCs in hemispheres from 10 to week- to 1-year-old mice were converted to common logarithm numbers. Subsequently, a reduction rate per week was calculated by a simple linear regression model (Prism 8 for Mac OS, ver. 8.4.3). The slope value (− 0.008362 ± 0.001760, mean ± SEM, *p* = 0.0006) was converted to a probability of NSC number reduction per week (10^-0.008362 = 0.98092996). An exponential curve of y = (0.98092996)^x starting with the 10-week value (948.7) was drawn by a tool provided by the GeoGebra web site (https://www.geogebra.org).

### Data analysis

Statistical analysis was performed using one-way or two-way ANOVA followed by Tukey’s or Dunn’s post hoc comparisons. If applicable, we used an unpaired two-tailed Mann–Whitney test or Fisher’s exact test. Normality of the data was analyzed by Shapiro–Wilk test. The level of significance was set at *P* < 0.05.

## Results

### GFP-expressing lentiviral labeling of NSCs

**Fig. 1:**
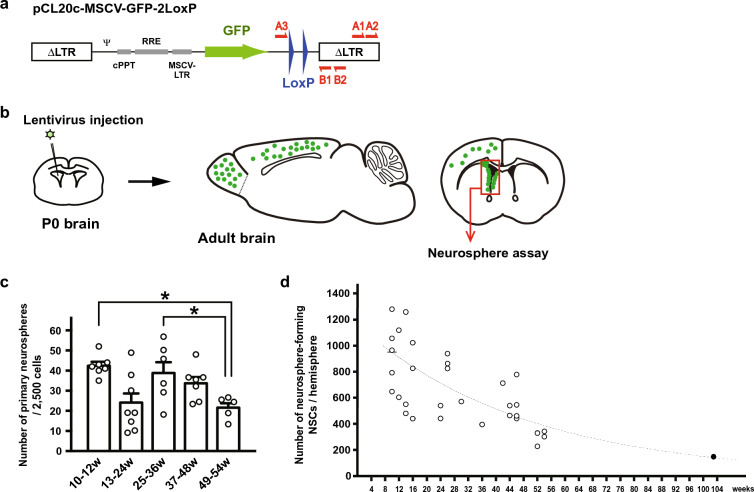
Lentivirus infection of neonatal mouse brains. **a** Structure of GFP-expressing lentivirus with two LoxP sequences and primers are shown. **b** Schematic diagram of lentiviral infection in neonatal mouse brains and analysis of adult brains. **c** Numbers of primary neurospheres generated from 2500 cells in the SEZ of the right hemisphere are shown. **d** Total numbers of neurosphere-forming NSCs in the right hemisphere over the lifespan are determined and shown along with an exponential graph (dotted line) that was calculated from the data between 10–54 weeks (open circles). A number of NSCs in a mouse at 104 weeks was overlaid on the graph (a closed circle). Data represent the mean ± SEM. *:* P* < 0.05 by one-way ANOVA followed by Tukey’s post hoc comparison

We first injected 1.0 × 10^5^ TU of GFP-2LoxP lentivirus (Fig. 1a) into the right ventricle of postnatal day (P) 0 mouse neonates as described previously [13]. The lateral and medial subependymal zone (SEZ) surrounding the right lateral ventricle of the mice were subjected to a colony-forming neurosphere assay [5, 21] (Fig. 1b). We determined the number of neurosphere-forming NSCs in the right SEZ and found that the number of primary neurospheres generated from 2,500 cells was significantly decreased in aged brain [*F*(4, 28) = 5.535, *P* = 0.0021; 10–12 weeks vs 49–54 weeks, *P* = 0.0085 and 25–36 weeks vs 49–54 weeks, *P* = 0.0487 by Tukey’s multiple comparisons test] (Fig. 1c). Similar to results in CD1 mice [13], the total number of NSCs gradually decline from 10 weeks to 1 year of age (948.7 ± 108.6 at 10 weeks and 322.6 ± 20.35 at 54 weeks, mean ± SEM per hemisphere) (Fig. 1d). Based on an assumption that the probability of NSC consumption per week *p*_total NSC_ is constant over time, a simple exponential regression model predicts the *p*_total NSC_ value as 0.01907, which is very similar to the results calculated from CD1 mice data (*p*_total NSC_ = 0.0189) [13]. We also estimated the total number of NSCs at 2 years of age by the equation *N*_103__weeks_ = 948.7 x (1–0.01907)^93^ = 158.3, which was very close to the 149.1 value obtained by the neurosphere assay (Fig. 1d).

### Depletion of transit amplifying cells by Ara-C treatment

**Fig. 2:**
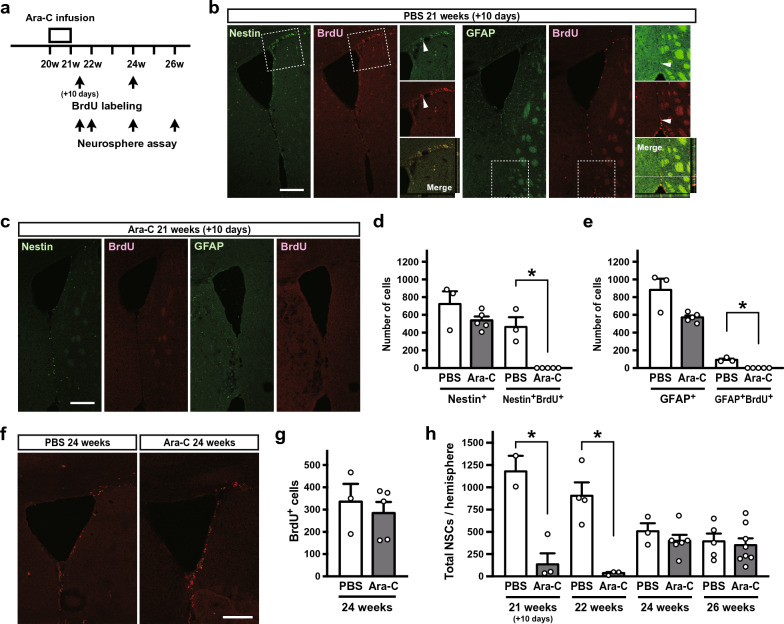
Ara-C infusion experiments. **a** Schematic diagram of the Ara-C infusion experiment. **b**, **c** BrdU incorporation into dividing cells surrounding the right lateral ventricle in coronal sections of the brains was determined by immunostaining. Immunostaining images for BrdU and either Nestin or GFAP of PBS- **b** or Ara-C- **c** infused brains 10 days after micro-osmotic pump insertion are shown. Areas within dashed boxes are shown at higher magnification at right. Arrowheads indicate double-positive cells. The merged pictures with orthogonal views of coronal z-stack images are also shown. **d**, **e** Numbers of Nestin^+^ **d** and GFAP^+^ **e** cells in the lateral and medial SEZ within 400 μm rostral to the crossing of the anterior commissure were counted. **f** Immunostaining for BrdU incorporation into dividing cells surrounding the right lateral ventricle in coronal sections of the brains 4 weeks after micro-osmotic pump insertion. **g** Total numbers of BrdU^+^ cells in the lateral and medial SEZ within 400 μm rostral to the crossing of the anterior commissure were determined. **h** A neurosphere assay was performed using subependymal cells from the brains 10 days, 2 weeks**,** 4 weeks, or 6 weeks after micro-osmotic pump insertion. Data were first analyzed by two-way ANOVA, which showed a significant interaction between Ara-C treatment and time course. Then, the data were analyzed by Kruskal–Wallis test. Data represent the mean ± SEM. Scale bar, 200 μm. *:* P* < 0.05 by Mann–Whitney test or by Kruskal–Wallis test followed by Dunn’s post hoc comparison

To investigate whether depletion of NSC progeny affects the quiescent NSC population, we used a well-established progenitor ablation paradigm [19, 20] (Fig. 2a). It was reported that vigorously dividing transit amplifying cells express Nestin and relatively quiescent NSCs express Nestin and GFAP [20]. We used a 13-h BrdU labeling protocol to label the entire transit amplifying cell population because these cells have a cell cycle time of approximately 12.7 h [22]. We found many Nestin^+^BrdU^+^ and a few GFAP^+^BrdU^+^ cells in the SEV of the PBS-infused hemispheres (Fig. 2b–e). On the other hand, the incorporation of BrdU into transit amplifying cells in the SEV was lost in the Ara-C-infused brain 10 days after the micro-osmotic pump insertion (Fig. 2c). As a result, Nestin^+^ cells showed a tendency to decrease in number in the Ara-C-infused brain (Nestin^+^ in PBS vs Ara-C, *P* = 0.5714 and Nestin^+^BrdU^+^ in PBS vs Ara-C, *P* = 0.0179 by Mann–Whitney test) (Fig. 2d). GFAP^+^BrdU^+^ cells were also significantly reduced in number in the Ara-C-infused brain (GFAP^+^ in PBS vs Ara-C, *P* = 0.0714 and GFAP^+^BrdU^+^ in PBS vs Ara-C, *P* = 0.0179 by Mann–Whitney test) (Fig. 2e). Total number of BrdU^+^ cells was recovered to a level comparable to controls 4 weeks after the insertion (*P* = 0.5714 by Mann–Whitney test) (Fig. 2f, g). Next, we determined the number of neurosphere-forming NSCs in the SEZ of the right hemisphere and found that the total number of NSCs in the Ara-C-infused hemispheres was drastically reduced 10 days or 2 weeks after the pump insertion as compared to those of PBS-infused hemispheres (*P* = 0.0068 by Kruskal–Wallis test; PBS vs Ara-C @10 days, *P* = 0.0297 and @2 weeks, *P* = 0.0027 by Dunn’s multiple comparisons test) (Fig. 2h). However, the number of NSCs was restored to control levels at 4 or 6 weeks although the number of NSCs in the PBS-infused hemispheres was slightly lower than the hemispheres of 24–26-week-old mice without pump insertion surgery (722.0 ± 97.1, mean ± SEM, *n* = 5) or calculated value by the equation described above [*N*_26 weeks_ = 948.7 x (1–0.01907)^16^ = 697.2].

**Fig. 3:**
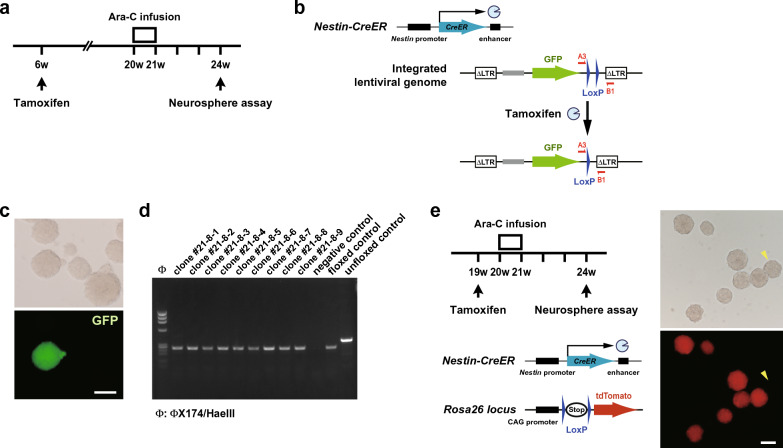
Tamoxifen injection and Ara-C infusion experiments. **a** Schematic diagram of the Ara-C infusion experiment following tamoxifen injection. **b** Tamoxifen induces recombination between two LoxP sequences within the lentiviral genome in *Nestin*-lineage cells. PCR primers to detect the recombination are shown. **c** Primary neurospheres including GFP^+^ neurospheres are shown. **d** DNA from amplified daughter neurospheres clonally derived from individual GFP^+^ spheres was subjected to recombination-detecting PCR. Unfloxed and floxed controls indicate PCR amplification from the lentiviral genome before and after recombination, respectively. **e** Schematic diagram of the Ara-C infusion experiment following tamoxifen injection using *Nestin-CreER*::Ai14 mice. Most of primary neurospheres generated 4 weeks after the Ara-C treatment expressed tdTomato. Arrowheads indicate a tdTomato^–^ neurosphere. Scale bars, 100 µm

The reduction of neurosphere formation shortly after the termination of Ara-C infusion could be due to a carry-over effect of Ara-C that was incorporated and remained within the cells unmetabolized or without excretion. The residual Ara-C in NSCs could kill the cells when stimulated to proliferate by growth factors in the culture. Alternatively, it is possible that Ara-C treatment eradicates substantial portion, if not all, of neurosphere-forming NSCs and that the NSC population is later replenished by dormant (deep quiescent) [20, 23] or even primitive NSCs [24]. To test the latter possibility, we induced tamoxifen-mediated recombination in *Nestin*-lineage NSCs (Fig. 3a, b) because those dormant or primitive NSCs are reported to scarcely express *Nestin*. Following tamoxifen administration at 6 weeks of age, Ara-C was infused into the right lateral ventricle through a micro-osmotic pump. Four weeks after the pump insertion, the neurosphere assay yielded a comparable number of neurospheres including lentivirus-infected GFP^+^ neurospheres (Figs. 2h, 3c). Subsequently, the GFP^+^ neurospheres were individually picked up and amplified in the passaging culture. DNA was extracted from the resultant daughter neurospheres and subjected to PCR to reveal that all clones (13 out of a total 13 GFP^+^ neurospheres from two lentivirus-injected *Nestin-CreER* mice) exhibited shorter DNA amplification from the cre-mediated floxed lentiviral genome (Fig. 3d). One may still argue that *Nestin*^+^ NSCs in the brain of 6-week-old mice could become *Nestin*^–^ dormant NSCs by the time of Ara-C treatment. To exclude this possibility, we utilized *Nestin-CreER*::Ai14 mice, in which tdTomato red fluorescent protein is expressed in *Nestin*-lineage cells after tamoxifen-mediated Cre recombination. We induced the recombination one week prior to the pump insertion and performed the neurosphere assay 4 weeks after the Ara-C infusion (Fig. 3e). Most of resultant primary neurospheres expressed tdTomato (Fig. 3e) and percentages of tdTomato^+^ neurospheres from Ara-C-infused brains were comparable to those from PBS-infused brains [87.829 ± 4.90% in PBS-infused (*n* = 4) and 88.64 ± 1.66% in Ara-C-infused (*n* = 3) brains, mean ± SEM). Thus, these results support the notion that *Nestin*-lineage quiescent NSCs survived the cytotoxicity of Ara-C for one week and restarted producing transit amplifying cells.

### Fate of NSCs after the depletion of transit amplifying cells

**Fig. 4:**
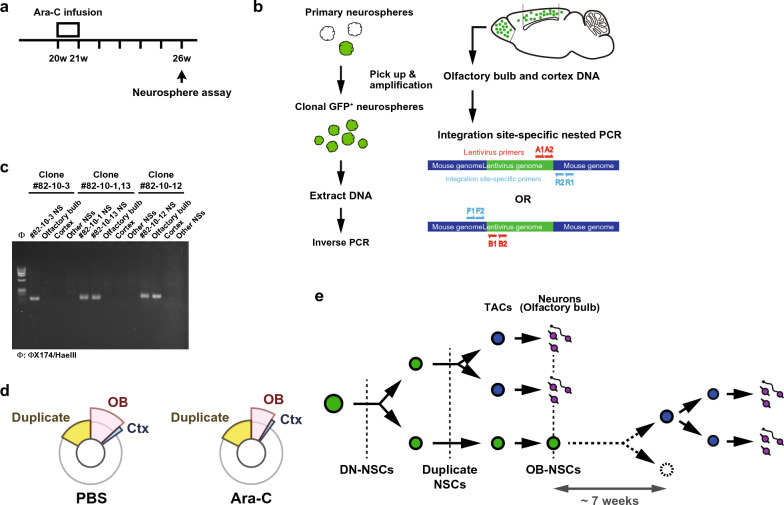
Detection of progeny from each NSC. **a** Schematic diagram of the Ara-C infusion, followed by the neurosphere assay. **b** Schematic diagram of the clonal isolation of lentivirus-infected NSCs and inverse PCR to identify the lentivirus integration sites. **c** DNA from each neurosphere clone, the olfactory bulb, and the cortex, and mixed DNA from the other neurosphere clones (Other NSs) derived from the same brain were subjected to integration site-specific nested PCR. Representative PCR results from a DN-NSC (left, Clone #82-10-3), duplicate NSCs (middle, Clones #82-10-1 and #82-10-13), and an OB-NSC (right, Clone #82-10-12) from the same brain of an Ara-C-infused mouse are shown. **d** Analysis of the 53 and 69 independent GFP^+^ neural stem cell clones derived from PBS- (left panel) and Ara-C-infused (right panel) mice, respectively, is shown. **e** Schematic diagram of our model. DN-NSCs undergo symmetric division to produce two daughter NSCs, which can be detected as duplicate NSCs until one or both progeny differentiate into transit amplifying cells (TACs) or die. After one daughter cell is consumed to provide olfactory bulb neurons, the other NSC can be detected as an OB-NSC until it is ultimately lost

We next investigated whether depletion of NSC progeny altered the behavior of quiescent NSCs by clonally tracing their fate as described previously [13]. We performed the neurosphere assay at a clonal cell density of 5 cells/µl in the culture, 6 weeks after the pump insertion (Fig. 4a). We could isolate 78 GFP^+^ neurospheres from four PBS-infused mice and 110 GFP^+^ neurospheres from eight Ara-C-infused mice. Single primary GFP^+^ neurospheres were individually picked up under a fluorescent microscope, mechanically triturated into single cells, and re-cultured using the same culture conditions. We extracted DNA from clonally amplified neurosphere clones and DNA was then subjected to inverse PCR to determine lentiviral integration sites as described previously [13] (Fig. 4b). DNA was also extracted from the olfactory bulb and cortex on the injected side of each mouse, from which GFP^+^ neurospheres were isolated. Among the 78 GFP^+^ neurosphere clones derived from PBS-infused mice and 110 GFP^+^ clones from Ara-C-infused mice, the integration site of the lentiviral genome was identified in 53 and 69 clones, respectively. By using an integration site-specific primer for the mouse genome together with a primer for the lentiviral genome, the presence of the lentiviral genome was determined by PCR in a clone-specific manner (Fig. 4c).

We hypothesized that if quiescent neurosphere-forming NSCs were killed in part by Ara-C treatment, this reduction should be later replenished by symmetric expansive divisions by NSCs themselves because the NSC population size was comparable between Ara-C-infused and control hemispheres after the cessation of Ara-C treatment and because all NSCs were *Nestin*-lineage (Figs. 2f, 3d, e). While most of the clone-specific PCR analyses amplified the expected length of DNA fragments exclusively from DNA of the original neurosphere clones and not from other clones derived from the same mouse brain, we found that 4 pairs of clones and one triplicated clone in the control brain (16.98%) and 6 pairs of clones in the Ara-C infused brain (17.39%) shared the same integration site (one example from the Ara-C clones is shown in Fig. 4c, middle panel), which revealed no statistically significant changes between the two groups by Fisher’s exact test (*P* > 0.9999) (Fig. 4d). Thus, our results suggest that Ara-C treatment hardly increases the possibility of symmetric expansive divisions of neurosphere-forming NSCs, supporting a notion that the apparent reduction of NSCs at 10 days or 2 weeks after the pump insertion was due to a carry-over effect of Ara-C that happened in the culture shortly after cessation of the Ara-C infusion.

We next determined the presence of lentiviral genomes in the olfactory bulb and the cortex in a clone-specific manner by nested PCR using 2 pairs of primers, one pair within the lentivirus and the other within the mouse genome near the integration sites (Fig. 4b). Most of the NSC clones (45 out of 53 in PBS-infused brains and 62 out of 69 in Ara-C-infused brains) contributed no detectable progeny to both regions [referred to as double negative (DN)-NSCs hereafter; one example is shown in Fig. 4c, left]. On the other hand, 7 NSC clones provided cells to the olfactory bulb (referred to as OB-NSCs) and one to the cortex (referred to as Ctx-NSCs) in PBS-infused brains and 6 clones contributed to the olfactory bulb and one to the cortex in Ara-C-infused brains (one example of OB-NSCs is shown in Fig. 4c, right). No NSC clones that produced progeny cells for both regions were evident. Thus, although Ara-C treatment killed most transit amplifying cells that were otherwise destined to migrate to the olfactory bulb, the percentage of OB-NSCs in the Ara-C brain showed only a marginal reduction as compared to that in control brain (13.21% in PBS-infused vs 8.70% in Ara-C-infused brain, *P* = 0.5560) (Fig. 4d). This is possibly due to amplification of transit amplifying cells, which are generated from NSCs by asymmetric division or by symmetric differentiating division, and the supply of olfactory bulb neurons through the rostral migratory stream lasts over several weeks [25]. If this is the case, Ara-C treatment during only the very early phase of amplification, when transit amplifying cells start providing postmitotic neuroblasts, would eradicate progeny and make otherwise OB-NSCs become counted as DN-NSCs.

## Discussion

In this study, we demonstrate that a one-week Ara-C infusion into the lateral ventricle eradicates most transit amplifying cells. In addition, substantial portion of quiescent NSCs would undergo a division during the 7-day Ara-C infusion since one study estimates their cell cycle time approximately 15 days or longer [25]. If one round of mitosis in the presence of Ara-C is enough to kill quiescent NSCs, this assumption predicts that surviving NSCs would increase symmetric divisions to replenish the reduction because the numbers of quiescent NSCs were comparable between Ara-C- and PBS-infused brains 3–5 weeks after the cessation of the infusion. However, we could detect no evidence for the increase of symmetric expansive divisions by quiescent NSCs in the Ara-C-infused brain: neither the increase of percentage of Duplicate-NSCs nor the emergence of four or more neurosphere clones that shared the same lentiviral integration sites. Since the sample size of our current study is relatively small, comprehensive studies on individual NSCs in the brain are awaited.

While dormant NSCs are considered mostly Nestin-negative and hardly detectable by the neurosphere assay [20, 23], it is formally possible that a few Nestin^+^ NSCs in this population respond to the reduction of NSCs and transit into quiescent, neurosphere-forming NSCs. Although this possibility remains to be investigated, the most likely scenario, we think, is that quiescent NSCs in the brain survive Ara-C incorporation by at least one round of mitosis and that the apparent reduction of neurosphere formation shortly after the Ara-C infusion is likely due to a carry-over effect of Ara-C, which kills the cells when stimulated to proliferate in the culture.

Several thousand new neurons are generated in the rodent olfactory bulb that are derived from neural precursors in the SEZ and functionally integrated into existing neural circuits [26, 27]. This neurogenesis system appears redundant because only a small portion of new neurons can survive for months and the rest of cells undergo apoptosis [28, 29]. The number of OB-NSCs could be, therefore, underestimated because our lentiviral integration site-specific nested PCR can detect only when 40 or more progeny of each NSC are present in the tissue [13]. However, we think this possibility quite remote because almost all OB-NSCs provide more than 100 progeny to the olfactory bulb [13]. The proportion of OB-NSCs is relatively constant: 13.70% at 4–6 weeks in a previous study [13] and 13.21% at 26 weeks in the PBS-infused brain in the current study. When considering that the number of progeny of each OB-NSC clone declines over time (6369 ± 1702 at 4–6 weeks and 3437 ± 1845 at 12 months, mean ± SEM in the previous study), these results suggest that the general fate of OB-NSCs is to be consumed. Given that the consumption rate of whole NSCs is 1.907% and supposing that most NSCs to be consumed (if not all) contribute to olfactory bulb neurogenesis prior to being lost, it is calculated that OB-NSCs persist approximately 7 weeks.

## Conclusion

Depletion of transit amplifying cells, immediate progeny of neural stem cells, did not induce symmetric expansive divisions of quiescent neural stem cells and did not recruit dormant neural stem cells to active neurogenesis. In addition, quiescent neural stem cells were gradually consumed after providing new neurons to the olfactory bulb.

## Data Availability

Data could be obtained upon request to the corresponding author.

## Abbreviations

NSC: Neural stem cell
SEZ: Subependymal zone
GFP: Green fluorescent protein
TU: Titer unit
LTR: Long terminal repeat
BrdU: 5-Bromo-2ʹ-deoxyuridine
Ara-C: Cytosine β-D-arabinofuranoside
SFM: Serum-free media
FGF-2: Fibroblast growth factor-2
EGF: Epidermal growth factor
GFAP: Glial fibrillary acidic protein
OB: Olfactory bulb
DN: Double negative
Ctx: Cortex

## References

[1] Altman J (1969). Autoradiographic and histological studies of postnatal neurogenesis. IV. Cell proliferation and migration in the anterior forebrain, with special reference to persisting neurogenesis in the olfactory bulb. J Comp Neurol.

[2] Spalding KL, Bhardwaj RD, Buchholz BA, Druid H, Frisén J (2005). Retrospective birth dating of cells in humans. Cell.

[3] Sanai N, Nguyen T, Ihrie RA, Mirzadeh Z, Tsai HH, Wong M, Gupta N, Berger MS, Huang E, Garcia-Verdugo JM, Rowitch DH, Alvarez-Buylla A (2011). Corridors of migrating neurons in the human brain and their decline during infancy. Nature.

[4] Bergmann O, Liebl J, Bernard S, Alkass K, Yeung MS, Steier P, Kutschera W, Johnson L, Landén M, Druid H, Spalding KL, Frisén J (2012). The age of olfactory bulb neurons in humans. Neuron.

[5] Reynolds BA, Weiss S (1992). Generation of neurons and astrocytes from isolated cells of the adult mammalian central nervous system. Science.

[6] Morshead CM, Reynolds BA, Craig CG, McBurney MW, Staines WA, Morassutti D, Weiss S, van der Kooy D (1994). Neural stem cells in the adult mammalian forebrain: a relatively quiescent subpopulation of subependymal cells. Neuron.

[7] Shen Q, Wang Y, Dimos JT, Fasano CA, Phoenix TN, Lemischka IR, Ivanova NB, Stifani S, Morrisey EE, Temple S (2006). The timing of cortical neurogenesis is encoded within lineages of individual progenitor cells. Nat Neurosci.

[8] Lim DA, Alvarez-Buylla A (2016). The adult ventricular-subventricular zone (V-SVZ) and olfactory bulb (OB) neurogenesis. Cold Spring Harb Perspect Biol.

[9] Gould E, Cameron HA, Daniels DC, Woolley CS, McEwen BS (1992). Adrenal hormones suppress cell division in the adult rat dentate gyrus. J Neurosci.

[10] Hayashi Y, Jinnou H, Sawamoto K, Hitoshi S (2018). Adult neurogenesis and its role in brain injury and psychiatric diseases. J Neurochem.

[11] Tropepe V, Craig CG, Morshead CM, van der Kooy D (1997). Transforming growth factor-α null and senescent mice show decreased neural progenitor cell proliferation in the forebrain subependyma. J Neurosci.

[12] Obernier K, Cebrian-Silla A, Thomson M, Parraguez JI, Anderson R, Guinto C, Rodas Rodriguez J, Garcia-Verdugo JM, Alvarez-Buylla A (2018). Adult neurogenesis is sustained by symmetric self-renewal and differentiation. Cell Stem Cell.

[13] Tanaka A, Ishida S, Fuchigami T, Hayashi Y, Kuroda A, Ikenaka K, Fukazawa Y, Hitoshi S (2020). Life-long neural stem cells are fate-specified at an early developmental stage. Cereb Cortex.

[14] Walsh C, Cepko CL (1992). Widespread dispersion of neuronal clones across functional regions of the cerebral cortex. Science.

[15] Tang F, Barbacioru C, Wang Y, Nordman E, Lee C, Xu N, Wang X, Bodeau J, Tuch BB, Sid-diqui A, Lao K, Surani MA (2009). mRNA-Seq whole-transcriptome analysis of a single cell. Nat Methods.

[16] Gerrits A, Dykstra B, Kalmykowa OJ, Klauke K, Verovskaya E, Broekhuis MJ, de Haan G, Bystrykh LV (2010). Cellular barcoding tool for clonal analysis in the hematopoietic system. Blood.

[17] Fuentealba LC, Rompani SB, Parraguez JI, Obernier K, Romero R, Cepko CL, Alvarez-Buylla A (2015). Embryonic origin of postnatal neural stem cells. Cell.

[18] Imayoshi I, Ohtsuka T, Metzger D, Chambon P, Kageyama R (2006). Temporal regulation of Cre recombinase activity in neural stem cells. Genesis.

[19] Doetsch F, Garcia-Verdugo JM, Alvarez-Buylla A (1999). Regeneration of a germinal layer in the adult mammalian brain. Proc Natl Acad Sci USA.

[20] Codega P, Silva-Vargas V, Paul A, Maldonado-Soto AR, DeLeo AM, Pastrana E, Doetsch F (2014). Prospective identification and purification of quiescent adult neural stem cells from their in vivo niche. Neuron.

[21] Hitoshi S, Kippin T, van der Kooy D, Seki T, Parent JM, Alvarez-Buylla A (2011). Culturing adult neural stem cells: application to the study of neurodegenerative and neuropsychiatric pathology. Neurogenesis in the adult brain II: clinical implications.

[22] Morshead CM, van der Kooy D (1992). Postmitotic death is the fate of constitutively proliferating cells in the subependymal layer of the adult mouse brain. J Neurosci.

[23] Belenguer G, Duart-Abadia P, Jordán-Pla A, Domingo-Muelas A, Blasco-Chamarro L, Ferrón SR, Morante-Redolat JM, Fariñas I (2021). Adult neural stem cells are alerted by systemic inflammation through TNF-α receptor signaling. Cell Stem Cell.

[24] Sachewsky N, Leeder R, Xu W, Rose KL, Yu F, van der Kooy D, Morshead CM (2014). Primitive neural stem cells in the adult mammalian brain give rise to GFAP-expressing neural stem cells. Stem Cell Reports.

[25] Morshead CM, Craig CG, van der Kooy D (1998). In vivo clonal analyses reveal the properties of endogenous neural stem cell proliferation in the adult mammalian forebrain. Development.

[26] Lois C, Alvarez-Buylla A (1994). Long-distance neuronal migration in the adult mammalian brain. Science.

[27] Biebl M, Cooper CM, Winkler J, Kuhn HG (2000). Analysis of neurogenesis and programmed cell death reveals a self-renewing capacity in the adult rat brain. Neurosci Lett.

[28] Winner B, Cooper-Kuhn CM, Aigner R, Winkler J, Kuhn HG (2002). Long-term survival and cell death of newly generated neurons in the adult rat olfactory bulb. Eur J Neurosci.

[29] Imayoshi I, Sakamoto M, Ohtsuka T, Takao K, Miyakawa T, Yamaguchi M, Mori K, Ikeda T, Itohara S, Kageyama R (2008). Roles of continuous neurogenesis in the structural and functional integrity of the adult forebrain. Nat Neurosci.

